# A New Medical Journal

**Published:** 1870-09

**Authors:** 


### A New Medical Journal.

We have received, from J. B. Lippincott & Co., publishers, the first number of the "*Medical Times*, "a semi-monthly Medical Journal, containing sixteen double-columns quarto pages of reading matter. The publishers announce a very formidable list of contributors, embracing the names of many very well known physicians of Philadelphia, and other cities, and the first number fully answers the highest expectations of the projectors of the enterprise.

